# Examining the Severity and Consequences of Patients Admitted With Acute Chest Pain via the Acute Physiology and Chronic Health Evaluation II Score and the Relevance of Adding High-Sensitivity Troponin Testing

**DOI:** 10.7759/cureus.97444

**Published:** 2025-11-21

**Authors:** Yasmeen Farag, Nada M Breika, Abdulmabod Omar, Mohamed E Owis, Mohamed Abdelrazek, Mina Soliman, Abdelrahman Embabi, Riham Nour, Muhamad Essam Muhamad Eldeeb, Mahmoud Farahat Ebrahim, Ahmad Adel Abdelhameed Muhammad, Ahmed G Ataalla, Amira Elzohary, Mohamed Tarek Saad Ramadan, Andrew Essam Habib Tawdros, Ahmed Maher Mohamed Elsayed, Nourhan Amr Mohamed Abouelsoud Salem

**Affiliations:** 1 Anesthesiology and Critical Care, Alexandria Faculty of Medicine, Alexandria, EGY; 2 Geriatrics, Peterborough City Hospital, Peterborough, GBR; 3 Clinical Pathology, Faculty of Medicine, Al-Azhar University, Cairo, EGY; 4 Laboratory Medicine, Hassan Ghazzawi Hospital, Abeer Medical Group, Jeddah, SAU; 5 Urology, Zagazig University, Zagazig, EGY; 6 Trauma and Orthopaedics, Mansoura University Hospitals, Mansoura, EGY; 7 Gastroenterology and Hepatology, Ain Shams University, Cairo, EGY; 8 Emergency Medicine, Royal Surrey NHS Foundation Trust, Guildford, GBR; 9 Emergency Medicine, University Hospitals of Birmingham, Birmingham, GBR; 10 Intensive Care Unit, Nasser Institute for Research and Treatment, Cairo, EGY; 11 Emergency Medicine, Royal Free London NHS Foundation Trust, London, GBR; 12 Emergency and Critical Care, Dr. Hassan Ghazzawi Hospital, Abeer Medical Group, Jeddah, SAU; 13 Cardiology, National Heart Institute, Cairo, EGY; 14 Emergency Medicine, Barnet Hospital, London, GBR; 15 Internal Medicine, Mansoura University, Mansoura, EGY; 16 Medicine, Beni Suef University, Beni Suef, EGY; 17 Cardiology, Benha Teaching Hospital, Benha, EGY; 18 Pediatric, Ain Shams University, Cairo, EGY

**Keywords:** acute chest pain, apache ii, critical care, high-sensitivity troponin, icu mortality, logistic regression, myocardial injury, prognostic accuracy, risk stratification, roc curve

## Abstract

Background

Acute chest pain is a critical presentation in intensive care units (ICUs), demanding rapid and accurate risk stratification to guide timely intervention. While the Acute Physiology and Chronic Health Evaluation II (APACHE II) score quantifies physiological severity, high-sensitivity (HS) troponin offers a biochemical measure of myocardial stress. However, their combined prognostic potential in ICU chest pain populations remains underexplored.

Objective

This study aimed to evaluate the severity and outcomes of ICU patients admitted with acute chest pain using the APACHE II score and to determine the added prognostic relevance of HS troponin testing for mortality prediction.

Methods

A cross-sectional analytical study of 100 ICU patients was conducted at Hassan Ghazzawi Hospital (January-August 2025). Data on demographics, physiological variables, APACHE II scores, and HS troponin status were analysed using SPSS version 26.0 (IBM Corp., Armonk, NY).

Results

Non-survivors exhibited significantly higher APACHE II scores (19.0 ± 6.2 vs 11.1 ± 6.1, p < 0.001). HS troponin positivity was strongly associated with mortality (87% vs 20%, p < 0.001; OR = 27.8). Both APACHE II (OR = 1.18) and HS troponin (OR = 21.7) were independent mortality predictors. The model demonstrated excellent discrimination (AUC = 0.89) and good calibration (p = 0.65).

Conclusion

Both APACHE II score and HS troponin levels were evaluated as independent predictors of mortality using multivariate logistic regression analysis. Combined physiological and biochemical assessment enables superior risk stratification, optimising early intervention and improving clinical decision-making.

## Introduction

Chest pain presentations are common in acute care settings, accounting for 5-9% of emergency department visits, though the exact prevalence among intensive care unit (ICU) admissions remains unclear due to limited data. Nevertheless, a substantial proportion of these patients are at high risk and require urgent assessment and intervention. Modern recommendations of the American Heart Association and the American College of Cardiology underline that clinical assessment, electrocardiography, and cardiac biomarkers should be integrated to enhance accuracy in diagnosing a patient and patient outcomes [1]. In this context, the physiologic severity scores and biochemical markers have become essential in the critical care prognostication.

The Acute Physiology and Chronic Health Evaluation II (APACHE II) score is an open-access score still among the most tested systems to determine the severity of the disease and prognosis of mortality of patients in intensive care. It gives a quantitative estimate of the likelihood of short-term survival by integrating acute physiological measures, age, and chronic health issues [2]. Even though it was initially designed to be used in general ICU groups, recent findings indicate that APACHE II can be applied to cardiac and mixed critical groups, where systemic compromise significantly affects outcomes, and that the score provides predictive ability for mortality in these populations (demonstrated by AUC values ranging from 0.74 to 0.80).

Simultaneously, with the advent of high-sensitivity cardiac troponin (HS-cTn) assays, the diagnostic and prognostic value of acute coronary syndrome has changed. Their high sensitivity of analytical methods allows them to detect the slightest myocardial damage, which is beneficial in the pre-disqualification or validation of an acute coronary attack [3]. It has been proven by clinical trials that faster algorithms could safely shorten decision-making and decrease the overcrowding of emergency departments [4]. Their diagnostic precision has been validated in practice, even in resource-constrained and high-volume clinical practice [5].

In spite of such improvements, there are still many ICU patients with acute chest pain who have high values of HS-cTn in the absence of overt infarction, indicating secondary myocardial stress, sepsis, or renal failure [6]. Chest pain and elevated troponin levels are relatively common among critically ill patients and may reflect a spectrum of underlying cardiac and systemic conditions. These findings are clinically relevant as they have been linked to adverse outcomes in intensive care settings. Therefore, this study was designed to evaluate the predictive ability of HS troponin and APACHE II scores for mortality in ICU patients, aiming to enhance early risk stratification and prognostic accuracy.

## Materials and methods

Study design and setting

This was an outcome-based observational study work in the ICU of Hassan Ghazzawi Hospital, which is a tertiary care facility in Jeddah, Saudi Arabia. The ICU serves mixed medical/surgery patients, and a large percentage of patients are admitted due to cardiovascular emergencies, such as acute chest pain, sepsis, and respiratory failure. A sample size of 100 patients was determined based on logistic regression requirements, ensuring at least 10 events per predictor variable (APACHE II score and HS-cTn status), which meets standard recommendations for model stability and reliability. The time frame was between January 2025 and August 2025, and all the qualified patients who got admitted within the time frame were included in the study. The study design allowed physiological severity and biochemical markers to be assessed at ICU admission to evaluate their relationship with early patient outcomes.

This study was approved by the Institutional Review Board (IRB) of Hassan Ghazzawi Hospital (IRB number: DHGH-MBEC-2508), and all procedures were conducted in accordance with national guidelines for human research.

Study population and eligibility criteria

One hundred patients were recruited by means of predetermined inclusion and exclusion criteria. The eligibility criteria included a sample size of adults aged 18 years and older who reported acute chest pain and were then hospitalised in the ICU to receive additional examination/treatment. There were male and female patients, so the sample was demographically representative. Patients who are moved to other ICUs, or those who have unfinished laboratory or clinical information within the initial 24 hours of admission, as well as patients who possess known chronic illnesses that might influence the interpretation of troponin (e.g., advanced chronic kidney disease or recent myocardial infarction), were not included. These criteria were used to make sure that cases were a new ICU admission with an assessable acute presentation.

Data collection and variables

The electronic medical records of the hospital were examined to get the data and checked with the physical ICU charts. Demographic data of every patient (age, sex), vital data (temperature, heart rate, respiratory rate), and laboratory data were gathered. The APACHE II scoring was actually computed in the first 24 hours of ICU stay and was computed under the normal standards that included twelve acute physiological variables, age points, and chronic health scores. The scores were then transformed into the predicted mortality percentage based on the APACHE II logistic regression equation, which was already determined [2]. Troponin I results were categorised as positive or negative according to the manufacturer’s reference limit [7]. Outcome was included as the end status of the patient in ICU stay, and it was either a non-survivor (died) or a survivor (discharged/transferred).

Data grouping

To facilitate comparative analysis, patients were stratified into two groups: Group 1: Patients who died in the ICU. Group 2: Patients who survived or were transferred to a step-down unit.

This classification enabled evaluation of both physiological severity (via APACHE II) and biochemical injury (via HS-cTn) in relation to mortality.

Statistical analysis

Data were analysed using SPSS version 26.0 (IBM Corp., Armonk, NY). Continuous variables were expressed as mean ± standard deviation (SD), while categorical variables were summarised as frequencies and percentages. The Shapiro-Wilk test was used to confirm the normal distribution of quantitative variables. Comparative analyses between survivors and non-survivors were performed using the independent-samples t-test for normally distributed continuous variables and the chi-square test for categorical variables such as HS troponin status and sex. The Pearson correlation coefficient was used to assess the linear relationship between the APACHE II score and predicted mortality percentage. To identify independent predictors of mortality, a binary logistic regression model was constructed with ICU outcome (survived/died) as the dependent variable, and APACHE II score and HS troponin status as independent predictors. Model calibration was assessed using the Hosmer-Lemeshow goodness-of-fit test, while discriminatory capacity was evaluated via the area under the receiver operating characteristic (ROC) curve. Statistical significance was established at a p-value < 0.05.

## Results

One hundred patients who were admitted to the ICU and presented with acute chest pain were factored into the analysis. Data integrity controls were used to ensure completeness of all variables, and the Shapiro-Wilk test was used to test the normality of continuous parameters. Analytical comparisons, correlations, and regression analysis were done as per the analytical plan that was formulated in advance.

Normality and descriptive statistics

The use of parametric methods was justified because all the continuous variables, including age, temperature, heart rate, respiratory rate, APACHE II Score, and APACHE II predicted mortality, on the one hand, were all normally distributed (p > 0.05 in Shapiro-Wilk tests). The mean age was 61.6 ± 15.5 years, and it was mostly male (63.0 -37.0). Our average temperature was 37.46 ± 0.85°C, heart rate was 107.8 ± 22.2 bpm, and respiratory rate was 24.9 ± 4.6 breaths/minute. The overall APACHE II score was 15.6 ± 7.3 with a mean predicted mortality of 26.0 ± 16.0% (Table 1).

**Table 1:** Group-wise descriptive statistics of continuous variables Max = maximum; Min = minimum; n = number; SD = standard deviation

Variable	Died (Mean ± SD)	Survived (Mean ± SD)	Average (Mean± SD)	Min	Max	n
Age (years)	63.8 ± 14.2	59.1 ± 16.8	61.6 ± 15.5	21	92	100
Temperature (°C)	37.5 ± 0.9	37.4 ± 0.8	37.46 ± 0.85	36.0	40.3	100
Heart Rate (bpm)	112.3 ± 21.1	102.3 ± 22.6	107.8 ± 22.2	30	170	100
Respiratory Rate (breaths/minute)	25.4 ± 4.9	24.2 ± 4.2	24.9 ± 4.6	15	33	100
APACHE II Score	19.0 ± 6.2	11.1 ± 6.1	15.6 ± 7.3	0	38	100
APACHE II Predicted Mortality (%)	33.5 ± 16.2	16.5 ± 9.7	26.0 ± 16.0	4	85	100

Group comparisons: survivors vs non-survivors

Independent-samples t-tests revealed statistically significant differences in APACHE II parameters between survivors and non-survivors (Table 2).

**Table 2:** Independent t-tests comparing survivors and non-survivors p-value < 0.05 = statistically significant

Variable	Mean (Died)	Mean (Survived)	t-statistic	p-value	Cohen’s d
Age (years)	63.8	59.1	1.61	0.111	0.30
Temperature (°C)	37.5	37.4	0.45	0.651	0.09
Heart Rate (bpm)	112.3	102.3	2.33	0.022	0.45
Respiratory Rate (breaths/minuter)	25.4	24.2	1.21	0.230	0.24
APACHE II Score	19.0	11.1	6.73	<0.001	1.12
APACHE II Predicted Mortality (%)	33.5	16.5	6.41	<0.001	1.08

Categorical associations are shown in Table 3.

**Table 3:** Association of categorical variables with ICU outcome p-value < 0.05 = statistically significant

Variable	Category	Died	Survived	χ²	p-value
HS Troponin	Positive	47	7	43.4	<0.001
	Negative	9	37		

The odds of death in patients with positive HS troponin compared with troponin-negative patients (Table 4).

**Table 4:** Odds ratio for HS troponin 95% CI for an OR

Measure	OR	95 % CI (Lower-Upper)
Troponin Positive vs Negative	27.8	10.5-73.8

Multivariable logistic regression analysis

Binary logistic regression was performed with ICU mortality (died = 1, survived = 0) as the dependent variable and APACHE II score and HS troponin status as predictors (Table 5).

**Table 5:** Binary logistic regression model predicting ICU mortality p-value < 0.05 = statistically significant OR (95 % CI) = 95% CI for an OR Coefficient (B) = unstandardized regression coefficient

Predictor	Coefficient (B)	OR (95 % CI)	p-value
APACHE II Score	0.165	1.18 (1.08-1.29)	<0.001
HS Troponin Positive	3.08	21.7 (5.9-79.5)	<0.001

The model achieved a McFadden pseudo-R² = 0.48, an AUC = 0.89, and an accuracy of 86 % at the 0.5 cut-off, demonstrating excellent discrimination. The Hosmer-Lemeshow test confirmed good calibration (χ² = 5.94, df = 8, p = 0.65), indicating no significant deviation between observed and predicted probabilities (Table 6).

**Table 6:** Model performance summary p-value < 0.05 = statistically significant

Metric	Value
McFadden pseudo-R²	0.48
Area Under ROC (AUC)	0.89
Classification Accuracy	86 %
Hosmer-Lemeshow χ²	5.94
df	8
p-value	0.65

## Discussion

The current article critically appraised the prognostic usefulness of the APACHE II score and high-sensitivity (HS) troponin testing in patients who arrived at the ICU with acute chest pain. The results showed that both markers were effective predictors of mortality, and a combination of the two markers was a more detailed estimate of risk. The ensuing discussion interprets these findings in terms of available literature, emphasising the clinical significance of combining physiological scoring systems with biochemical biomarkers, and presenting the research findings in the context of the modern critical care and cardiovascular guidelines.

Summary of key outcomes

A total of 100 patients admitted to the ICU was used to test the appropriate relationship between prognostic variables and physiological severity and biochemical markers. The mean age of the cohort was 61.6 ± 15.5 years, and 63% of the cohort was male and 37% female. The total mortality stood at 56 %, which makes this group of patients very critical. Such continuous variables as age, temperature, heart rate, respiratory rate, APACHE II score, and the predicted mortality percentage were shown to have a normal distribution according to a Shapiro-Wilk test, which confirmed the application of parametric statistical analysis. The average APACHE II score was 15.6 ± 7.3, which gave an average predicted death rate of 26.0 ± 16.0%.

Comparison between survivors and non-survivors showed a high difference in physiological and biochemical factors. The APACHE II scores (19.0 ± 6.2 vs. 11.1 ± 6.1, p < 0.001) and the percentages of mortality predicted (33.5 ± 16.2 vs. 16.5 ± 9.7, p < 0.001) were significantly higher in the non-survivors. Heart rate was also slightly higher in the non-survivor group, among other key parameters, with age, temperature, and respiratory rate being statistically insignificant. The test of HS troponin showed a high association with outcome; 54% of all patients were found to have positive troponin, and mortality was 87% among the group of positive troponin patients versus 20% with negative troponin. The chi-square test proved that there was a very significant relationship between HS troponin positivity and death (p = 0.001), and an odds ratio of 27.8 (95% CI: 10.5-73.8). There was no significant impact of sex distribution.

The APACHE II score and the predicted mortality percentage displayed a strong positive correlation (r = 0.92, p < 0.001), indicating the internal validity of the APACHE scoring system among this group of critically ill patients. Binary logistic regression analysis showed that APACHE II score and screened HS troponin status were independent predictors of ICU mortality. Every point higher in APACHE II score elevated the chance of death by one-fifth (OR = 1.18, 95% CI: 1.08-1.29, p < 0.001), and the HS troponin positivity increased the risk of death more than twenty times (OR = 21.7, 95% CI: 5.9-79.5, p < 0.001) independently. The multivariate model had a great level of discrimination with an area under the ROC curve (AUC) of 0.89, a McFadden pseudo-R2 of 0.48, and a total classification accuracy of 86%. Hosmer-Lemeshow test showed good calibration (χ² = 5.94, df = 8, p = 0.65), which is one of the indicators that the probabilities forecasted were in close agreement with the observed ones.

Overall, both systemic physiological derangement and biochemical results of myocardial injury had a significant impact on the mortality of ICU patients presenting with acute chest pain. The combination of a validated physiological scoring system and a cardiac biomarker was statistically significant and did not depend on other factors to predict death, which showed that physiological scores combined with a cardiac biomarker give better prognostic value. These results highlight the clinical significance of integrated physiological and biochemical analysis to inform the clinical decisions of early risk stratification and risk management in severely ill patients with acute chest pain.

The high predictive accuracy of HS troponin in this data agrees with the dynamic view of cardiac biomarkers in critical care. HS troponin has been identified as the biomarker of choice in diagnosing and stratifying acute coronary syndromes because it is better than traditional assays, both in terms of sensitivity and early detection. The positive HS troponin in the current cohort showed almost 28 times higher mortality risk compared to the negative group, which proved that it has a prognostic value in addition to diagnostic classification. This is in line with Sandoval et al., who have indicated that even small increases in troponin, as was identified by HS assays, have independent prognostic value for adverse cardiovascular events [1].

Notably, not every troponin increase is symptomatic of acute myocardial infarction (AMI). Geladari et al. pointed out that HS troponin is an indicator of cardiac injury regardless of its aetiology, like sepsis, kidney failure, and critical illness, which are all pertinent in the ICU setting [8]. This wide diagnostic range justifies the present observation that even in the absence of myocardial infarction as the leading diagnosis, HS troponin can serve as a mortality prediction. Furthermore, the findings build upon the previous studies by Mokhtari et al., who showed that early risk identification can be improved with the help of rapid troponin-based algorithms, including the European Society of Cardiology (ESC) zero-/one-hour rule-out protocol, and the diagnostic safety is preserved [4]. Even though the mentioned trial examined the emergency department populations, the current findings indicate the same predictive capability with troponin used in ICU risk stratification models.

The level of HS troponin interpretation of critically ill patients should be taken with caution, as it can be caused by many factors of stress rather than by direct coronary arrest. The fact that troponin-positive individuals die at a higher rate in the current study is consistent with the results of large epidemiologic studies, which indicate that high levels of troponin are associated with general physiologic worsening and dysfunction of different organs as opposed to cardiac pathology [1].

This increased prognostic function is supported by recent evidence. Chen et al. determined HS troponin T-specific cut-offs according to kidney functions, underlining that non-ischemic increases in renal impairment continue to predict adverse outcomes [7]. Likewise, Geladari et al. explained chronic kidney disease as an environment in which continuously high troponin values are an increased cardiovascular risk, and not analytic noise [8]. These results put the existing findings in perspective, whereby in ICU environments, when other comorbidities like sepsis, hypoxia and renal dysfunction coexist, troponin is not a specific indicator of infarction but is a global biomarker of tissue injury and risk of mortality.

In that sense, the fact that the finding of troponin positivity correlates with mortality in the current dataset allows a continuum of injury model. Troponin increase can be used to determine the patients whose cardiovascular capacity is overwhelmed by systemic stressors, which is the reason why it remains a predictive variable even after the APACHE II score has been considered. Therefore, to identify stress is not only a diagnostic tool but also a physiological multi-organ stress integrator, which is in line with current cardiology and critical care views [9].

Although the APACHE II scoring system was initially designed to be used with general ICU populations, it is one of the most tested systems in predicting mortality in critical illness [10,11]. The fact that higher APACHE II scores were relevant to death (mean 19.0 vs. 11.1, p < 0.001) in the present study supports the earlier validation studies in various clinical settings. Vandenbrande et al. established that APACHE II continues to provide predictive value in cohorts of ICU patients in the pandemic, with a high degree of discrimination (AUC = 0.80 or more) of mortality prediction [12]. Similarly, Fernandes et al., Vandenbrande et al., and Ali et al. demonstrated its consistency in both viral pneumonia and sepsis and reported that the exposure of accumulating physiological derangement is not single-organ failure [13-15].

The predictive quality of APACHE II in the current setting highlights the general quality of acute chest pain manifestations that necessitate hospital admission to the ICU. Most of such patients do not have isolated coronary events but rather multi-system, shock, respiratory distress or renal failure. Thus, the APACHE II score is used to supplement the information provided by the HS troponin, and it measures total physiological load. It is in line with a study by Bellino et al., Cirik et al., and Zhang et al., who showed that simultaneous use of APACHE II and other clinical indices enhances the mortality risk assessment in sepsis [16-18]. The reliability of APACHE II as a generalised severity measure is supported by the consistency of predictive models across disease conditions.

The combination of APACHE II scores and HS troponin testing gives a complete prognostic model of ICU patients with acute chest pain. The logistic regression outcomes showed that every point increase in APACHE II was associated with an 18-fold increase in the likelihood of death, whereas positivity of troponin was associated with a risk over 20-fold, and the model had an excellent discrimination (AUC = 0.89). This relationship between physiological and biochemical assessment is a present-day trend of critical care assessment, in which no single measure is sufficient to represent the outcome risk.

Similar trends have been cited in heart studies. Rohde and colleagues have determined that HS troponins are interesting in risk stratification based on the extent of atherosclerotic vascular disease, and this is why they should be used as universal biomarkers of heart disease susceptibility [19]. On the same note, Cyon et al. found higher levels of troponin in acute kidney damage to predict mortality despite not having myocardial infarction, indicating that biochemical markers offer an additional contribution to physiological scores by themselves [20]. Combining these findings, the current findings support the clinical importance of dual-parameter prognostication, in which troponin is the measure of myocardial stress and APACHE II is the measure of systemic dysfunction.

Moreover, other research works like Cirik et al. compared various ICU scales and found APACHE II as one of the most powerful mortality variables alongside laboratory factors [17]. This is in line with the conceptual framework of the analysis at hand, whereby physiologic and biochemical measurements are combined to enhance the accuracy. The large McFadden pseudo-R2 (0.48) and calibration performance herein achieved testify to the strength of this model to use in a real-world ICU. The results are also consistent with the changing ESC and American Heart Association (AHA)/American College of Cardiology (ACC) guidelines. The 2023 ESC recommendations support the use of early troponin testing and combined clinical algorithms in order to enhance the predictability of outcomes at the presentation of acute coronary syndrome [21]. The current analysis gives reason to believe that this integration can still be useful even in post-admission to the ICU, with the death determinants no longer being limited to coronary occlusion but also including systemic instability.

Furthermore, Li et al. have shown that the ESC zero-/one-hour troponin algorithm could be diagnostic in busy emergency rooms and proved to be reliable in the real-world setting [22]. The present research will carry on this relevance in practice by demonstrating that it has prognostic value in a critically ill group. It further supplements the results obtained by Dallmeier et al., who stated that longitudinal variations in the levels of HS troponin are associated with overall mortality for three years, even in non-cardiac patients [10]. All this evidence indicates that troponin is no longer a diagnostic biomarker, but a continuous prognostic agent, which the results of the current study also prove. With a pathophysiological perspective, the relationship between elevated troponin and mortality can be explicated as an indicator of worldwide myocardial load. Sustained tachycardia, hypoxemia, and systemic inflammation are some of the factors that hasten myocardial oxygen imbalance, leading to subclinical necrosis in response to HS troponin assays [3]. This process clarifies the presence of a mortality gradient even in the non-confirmed patients of myocardial infarction.

Moreover, the systemic physiology and cardiac dysfunction interaction measured by APACHE II demonstrates the significance of multi-organ evaluation. As an example, Peng et al. described that the lower level of physical performance, measured in slower walking speed, was linked to an increased risk and mortality of cardiovascular disease in older adults [6]. These results help to endorse the idea that cardiovascular outcomes are interdependently related to the general physiological resilience. Therefore, the current findings support the need to implement multidisciplinary strategies, which integrate cardiological, nephrological, and intensive care views in the acute chest pain treatment in the critical environment.

The applied implications of these findings are very significant. To start with, APACHE II scoring should be incorporated into the baseline of the initial evaluation of ICU patients with chest pain, as it will offer an objective risk level and resource allocation. Second, early HS troponin testing helps clinicians identify patients at high risk, prompting more attention or increased care. Third, the integrated model may be used to derive prognostic counselling and decisions about invasive interventions, especially in situations where such decisions must be made using limited resources; resource prioritisation is needed.

Multidisciplinary protocols that align emergency, cardiology and critical care services are also a recommendation of these results. The 2021 AHA/ACC guideline solemnly recommends the use of the so-called integrated diagnostic pathways combining laboratory biomarkers with clinical scoring systems to maximise the results [1]. The current research can validate such a suggestion, and quantifiable data of a better prognostic accuracy can be presented in case of such integration used in ICU practices. Also, based on the predictive capabilities, the introduction of these markers into electronic health systems to generate automatic mortality warnings could improve situational awareness among clinicians. The quality model calibration proves that it can be used in real-time clinical decision tools.

Strengths of the study

This study’s primary strength lies in its integration of physiological and biochemical predictors, APACHE II scores, and HS troponin to assess mortality risk in ICU patients with acute chest pain. This combined approach provides a more comprehensive evaluation of patient status, capturing both systemic physiological derangement and myocardial injury. Methodologically, the study employed clearly defined inclusion criteria, rigorous data validation, and robust statistical analyses, including logistic regression and model calibration. Additionally, both APACHE II and HS troponin are widely accessible and feasible for implementation in diverse clinical settings, enhancing the practical applicability of the findings for early risk stratification and clinical decision-making.

Future directions

Future research should aim to confirm these observations in larger, multicentre prospective studies to enhance the generalizability of the findings across diverse ICU populations. The use of serial HS troponin measurements could provide insights into temporal changes and support dynamic prognostic modelling. Incorporating additional biomarkers, such as lactate, inflammatory markers, or natriuretic peptides, may further refine predictive accuracy when combined with APACHE II scores. Comparative studies evaluating the performance of other scoring systems (e.g., SOFA, SAPS II) alongside troponin could help identify the most effective hybrid models for critical care prognostication. Additionally, the integration of combined APACHE II and HS troponin assessment into electronic health systems could potentially facilitate real-time mortality alerts and support clinical decision-making. Finally, interventional studies are warranted to determine whether management strategies guided by these combined risk profiles can improve survival in ICU patients presenting with acute chest pain.

Limitations

This study has several limitations that should be considered when interpreting the findings. First, the sample size was relatively small and drawn from a single centre, which may limit the generalizability of the results to broader ICU populations. Second, troponin was analysed as a categorical variable, which may have reduced the precision of its prognostic value; evaluating troponin as a continuous variable and determining optimal cut-off points for mortality could provide additional insight. Third, important clinical variables were not included, such as SOFA scores, hemodynamic instability, the use of vasopressors, sepsis, or other comorbidities, which may influence mortality and confound associations. Finally, the observational design precludes causal inference, and further studies incorporating these variables and larger, multicentre cohorts are needed to validate and refine the prognostic utility of combining APACHE II scores with HS troponin.

## Conclusions

In this study, APACHE II scores and HS troponin were associated with mortality in ICU patients presenting with acute chest pain. The combination of physiological severity assessment and biochemical markers provided a more comprehensive evaluation of patient risk than either measure alone. Higher APACHE II scores reflected greater systemic physiological derangement, while positive HS troponin indicated myocardial stress or injury, even in the absence of acute infarction. A multivariable model incorporating both parameters demonstrated good discrimination and calibration for mortality prediction. These findings suggest that integrating physiological and biochemical assessments may support early risk stratification, guide clinical management, and inform resource allocation in critically ill patients with acute chest pain.
